# Which patients with symptoms should be referred urgently for investigation of suspected cancer?

**DOI:** 10.1136/bmjonc-2024-000640

**Published:** 2024-11-21

**Authors:** Sarah F Moore

**Affiliations:** 1 University of Exeter, Exeter, UK

**Keywords:** Epidemiology

 Although asymptomatic screening has changed the way in which a few cancers are diagnosed, most patients diagnosed with cancer still present with symptoms. In the UK and many other countries, such as Denmark and Australia, most of these patients present in primary care. This poses a difficult question to the primary care physician who must try to tease out which patients with a particular symptom should be further investigated for serious underlying causes, including cancer. In the UK, there have been attempts to support those making these decisions, not least the introduction of national guidance on who should be referred for urgent investigation for cancer.^1^ Although this guidance has been shown to have had an impact on improving cancer outcomes for patients presenting with symptoms in primary care, there is still a long way to go to reach the National Health Service long-term plan goal of diagnosing 75% of cancers at an early stage by 2028.^2 3^ Changes to guidance and referral practices could play an important role in achieving this target.

One of the key limitations of current guidance is the lack of stratification of risk, even by basic measures such as age, sex and smoking status. The article by Barclay *et al* attempts to address this issue by providing a comprehensive evaluation of two key outcomes in a UK based cohort; cancer (at any site) or non-cancer death of patients presenting with one of 15 key symptoms in primary care.^4^ They report these outcomes, stratified by age, sex and smoking status and compare them to the outcomes for a representative sample of patients not selected for symptomatic presentation.

The current threshold for referral for urgent investigation is that a symptom should have a positive predictive value of 3% or more for a diagnosis of cancer at a specific site for referral to take place. One of the main findings in Barclay *et al* is that while for some symptoms patients do not exceed the current threshold for referral for *individual* cancer sites, a symptomatic patient’s overall risk of having an undiagnosed cancer at *any* site exceeds 3% at a much earlier age than might have been expected.^4^ This has implications for referral practices, which are generally focused on individual sites rather than overall risk associated with symptoms. In recent years, non-specific symptom pathways have been introduced which have begun to address this issue with some success; however, findings from this paper suggest there may be scope to extend their reach into symptoms such as abdominal pain that have traditionally been siloed into individual cancer site referrals.^5^

Another key finding in Barclay *et al* is that for some older patients, their risk of death from a non-cancer cause in the 12 months following a symptom is higher than their risk of being diagnosed with cancer.^4^ This competing risk of mortality is not currently addressed in guidance. Indeed, a recent systematic review highlighted a lack of certainty on how to manage older adults with these symptoms, with evidence of an association between increasing age and deferral of decisions to investigate for cancer.^6^ The findings of the study by Barclay *et al* contribute to the knowledge around risk in older adults, which could allow for more informed shared decision-making between patients, their families and healthcare professionals when deciding whether to pursue urgent referral to investigate for cancer.

The design of the study by Barclay *et al* brought many strengths, but there were inevitable limitations, which are clearly laid out in the main article and appendix 7.^4^ Some limitations are common to studies using coded data from electronic healthcare records, including potential missing data through lack of coding and difficulty with nuanced measuring of metrics such as smoking status. Other limitations included not making use of the longitudinal nature of the records and restriction of the number of symptoms investigated. However, while there are many other aspects that could have been explored, for example, risk of death following cancer diagnosis, the scope of the study was already so broad, and the information contained within it so comprehensive that these questions might be best served by further studies focused specifically on these areas.

The results of this study and of future studies adopting similar methodology have significant potential to be used in refining guidance for urgent cancer referrals, resulting in a better targeted and more effective process that supports patients and their doctors in well-informed shared decision-making. While we await these changes, doctors should feel empowered to use results from studies such as this to inform their practice, with the ultimate aim as always of improving outcomes for their patients.

## Data Availability

Data sharing not applicable as no datasets generated and/or analysed for this study.
